# A novel autoantibody signatures for enhanced clinical diagnosis of pancreatic ductal adenocarcinoma

**DOI:** 10.1186/s12935-023-03107-1

**Published:** 2023-11-16

**Authors:** Tiandong Li, Junfen Xia, Huan Yun, Guiying Sun, Yajing Shen, Peng Wang, Jianxiang Shi, Keyan Wang, Hongwei Yang, Hua Ye

**Affiliations:** 1 College of Public Health, Zhengzhou University, 450001 Zhengzhou, Henan Province China; 2 Henan Key Laboratory of Tumor Epidemiology and State Key Laboratory of Esophageal Cancer Prevention & Treatment, Zhengzhou University, 450052 Zhengzhou, Henan Province China; 3 Office of Health Care, The Third Affiliated Hospital of Zhengzhou University, 450052 Zhengzhou, Henan Province China; 4 Zhengzhou University, 450001 Zhengzhou, Henan Province China; 5 Henan Institute of Medical and Pharmaceutical Sciences, Zhengzhou University, 450052 Zhengzhou, Henan Province China; 6 Department of Gastrointestinal Surgery, The First Affiliated Hospital of Zhengzhou University, 450052 Zhengzhou, Henan Province China

**Keywords:** Pancreatic ductal adenocarcinoma, Autoantibody, Diagnosis, Immunodiagnostic, Model

## Abstract

**Background:**

Pancreatic ductal adenocarcinoma (PDAC) is a devastating disease that requires precise diagnosis for effective treatment. However, the diagnostic value of carbohydrate antigen 19 − 9 (CA19-9) is limited. Therefore, this study aims to identify novel tumor-associated autoantibodies (TAAbs) for PDAC diagnosis.

**Methods:**

A three-phase strategy comprising discovery, test, and validation was implemented. HuProt™ Human Proteome Microarray v3.1 was used to screen potential TAAbs in 49 samples. Subsequently, the levels of potential TAAbs were evaluated in 477 samples via enzyme-linked immunosorbent assay (ELISA) in PDAC, benign pancreatic diseases (BPD), and normal control (NC), followed by the construction of a diagnostic model.

**Results:**

In the discovery phase, protein microarrays identified 167 candidate TAAbs. Based on bioinformatics analysis, fifteen tumor-associated antigens (TAAs) were selected for further validation using ELISA. Ten TAAbs exhibited differentially expressed in PDAC patients in the test phase (*P* < 0.05), with an area under the curve (AUC) ranging from 0.61 to 0.76. An immunodiagnostic model including three TAAbs (anti-HEXB, anti-TXLNA, anti-SLAMF6) was then developed, demonstrating AUCs of 0.81 (58.0% sensitivity, 86.0% specificity) and 0.78 (55.71% sensitivity, 87.14% specificity) for distinguishing PDAC from NC. Additionally, the model yielded AUCs of 0.80 (58.0% sensitivity, 86.25% specificity) and 0.83 (55.71% sensitivity, 100% specificity) for distinguishing PDAC from BPD in the test and validation phases, respectively. Notably, the combination of the immunodiagnostic model with CA19-9 resulted in an increased positive rate of PDAC to 92.91%.

**Conclusion:**

The immunodiagnostic model may offer a novel serological detection method for PDAC diagnosis, providing valuable insights into the development of effective diagnostic biomarkers.

**Supplementary Information:**

The online version contains supplementary material available at 10.1186/s12935-023-03107-1.

## Introduction

Pancreatic ductal adenocarcinoma (PDAC) is a highly lethal malignancy, ranking third and fourth as the leading cause of cancer-related mortality, with an overall survival rate of approximately 10% [1–4]. The low survival rate can be primarily attributed to the difficulty in early diagnosis, which results in less than 25% of patients being eligible for curative surgical resection at the time of diagnosis [4]. Early diagnosis of PDAC has been shown to improve 5-year survival to 30% or more, highlighting the importance of early detection [5].

Tumor-associated autoantibodies (TAAbs) have emerged as promising biomarkers for the early diagnosis of cancer [6]. TAAbs are produced by the sera of cancer patients against tumor-associated antigens (TAAs) and can be detected months to years before diagnosis [6–8]. While numerous TAAbs have been studied in the detection of various cancers, including lung cancer [9], esophageal squamous cell carcinoma [10], and colorectal cancer [11], research on TAAbs for pancreatic cancer is relatively limited. EarlyCDT-Lung, which includes six TAAbs (anti-p53, anti-NY-ESO-1, anti-CAGE, anti-GBU4-5, anti-Annexin 1, and anti-SOX2), has been successfully applied in practice and has played a significant role in screening high-risk lung cancer groups [12]. Compared to other biomarkers like cell-free DNA and circulating tumor cell (CTC), TAAbs possess advantages including early emergence, persistence, stability, and easy detection [7, 13].

Currently, PDAC diagnosis primarily relies on imaging techniques such as CT, MRI, US, PET, and EUS; However, these options are costly and invasive for individuals, and diagnoses of the disease are usually made in late-stage [14]. Although carbohydrate antigen 19-9 (CA19-9) is extensively used as a serum biomarker in the clinical setting, its predictive value for accurate cancer detection is unsatisfactory, with 80% sensitivity and 75% specificity [15, 16]. Both diagnostic methods in clinical practice are unsatisfactory; there is a crucial need to identify novel biomarkers or investigate effective strategies to enhance the diagnostic accuracy of CA19-9.

In this study, we utilized human protein microarray technology to identify potential TAAbs. This high-throughput method enabled the comprehensive detection of TAAbs, allowing for cost-effective screening of valuable biomarkers for cancer diagnosis [17–21]. Additionally, we developed a robust immunodiagnostic model that can significantly enhance the detection capacity of CA19-9. We anticipate that this model will improve the early detection rate of PDAC, ultimately leading to better patient outcomes.

## Materials and methods

### Human serum samples

This study was comprised of 526 serum samples from the Biological Specimen Bank of Henan Key Laboratory of Tumor Epidemiology (Henan, China). Patients with PDAC and benign pancreatic diseases (BPD, including chronic pancreatitis, low grade intra- ductal papillary mucinous neoplasm, well-differentiated neuroendocrine tumor, solid pseudopapillary neoplasm, mucinous cystic neoplasm, serous cystadenoma and pseudocyst) were collected between August 2016 and September 2022 from three different hospitals, and normal controls (NC) were matched to cases by sex and age (± 5 years) from the healthy physical examination population. All blood samples were prepared according to standard protocol [22]. Briefly, 5 mL blood was drawn with an EDTA tube, then centrifuged at 3000 rmp for 5 min, the supernatant was transferred to enzyme-free Eppendorf 1.5 mL tubes and 200 uL of each tube was stored at -80 °C until use, avoiding repeated freeze-thaw cycles. The TNM staging criteria were identified based on the eighth edition of the American Joint Committee on Cancer (AJCC) staging system. All human participants have signed informed consent, and the study was approved by the Institutional Review Board of Zhengzhou University (ZZURIB2019001).

The inclusion criteria for PDAC patients in this study were highly stringent to ensure the accuracy and reliability of our results. Specifically, eligible participants met the following criteria: (1) a pathological diagnosis of PDAC; (2) the absence of autoimmune diseases; (3) no history of pancreatitis; (4) no history of other malignancies; and (5) all serum samples collected from PDAC patients were obtained prior to any treatments or surgery, ensuring that the samples were newly diagnosed. Additionally, all serum samples were absence of hemolysis or any visible precipitate in the serum samples before further detection.

### Serum TAAb profiling on HuProt™ protein arrays

Comprehensive profiling of serum TAAbs was conducted using HuProt™ Human Proteome Microarray v3.1, which contained 21,216 unique proteins. The microarrays were provided by CDI Laboratories and purchased from BC Biotechnology Co., LTD (Guangzhou, China). We applied 49 serum samples from the discovery phase to the HuProt™ arrays and detected autoantibody signals in 10 pooled PDAC and 10 pooled NC serum samples, which included 27 PDAC patients and 22 NCs. Samples were pooled according to age and gender to ensure uniformity. For the PDAC group, seven pooled samples were created by mixing every three sera, and three pooled samples were formed by mixing every two sera. As for the NC group, six pooled samples were generated by mixing every three sera, while the remaining four samples were used individually. The serum samples were diluted in 1:200 in binding buffer (1% BSA in PBST), following the experimental protocols used in our previous studies [17, 23].

### Identification of candidate TAAs based on HuProt™ protein microarray and bioinformatics

Firstly, TAAb signals were detected, normalized, and quantified after serum incubation on the HuProt™ arrays. Next, the priority autoantibodies were identified by following criteria: (1) statistical differences between cases and controls (*P* < 0.05), using the Mann-Whitney U test; (2) Fold change (FC) ≥ 1.2; and (3) positive rate (PR) of PDAC ≥ 50% while that of NC ≤ 10%. Then, Gene Ontology (GO) term enrichment analysis was performed to explore the significantly enriched pathways, and TAAs associated with immune biological processes were identified as potential biomarkers of interest. Finally, RNA-Seq data from UCSC Xena (https://xenabrowser.net/datapages/) was collected to assess the gene expression levels of candidate significant TAAs.

### Recombinant proteins and the detection of TAAbs by ELISA

Seven proteins (DBNL, HEXB, OSCAR, TRIM21, BNIP3L, LTF, and SLAMF6) were purchased from CUSABIO (Wuhan, China), and six proteins (FUCA2, GLB1, PSMD2, TXLNA, RAC1, and LILRB2) were purchased from Cloud-clone Corporation (Wuhan, China). Two recombinant proteins (p53/TP53 and p62/IGFBP2) were purified from our laboratory. The concentration, purity, and molecular weight of all proteins were confirmed using SDS/PAGE gel. The coating concentrations for the enzyme-linked immunosorbent assay (ELISA) were 0.125 ug/mL for FUCA2, LTF, RAC1 and 0.25 ug/mL for DBNL, GLB1, HEXB, OSCAR, PSMD2, TXLNA, TRIM21, BNIP3L, SLAMF6, LILRB2, TP53, IGFBP2. The details of the ELISA detection were described in our previous paper [18]. Briefly, each protein was coated on an ELISA plate overnight at 4 °C and incubated in 2% BSA at 37 °C for 2 h. Serum samples diluted at 1:100 in binding buffer (1% BSA in PBST) were then added to the ELISA plate and incubated at 37 °C for 1 h. After washing with PBST, the plates were incubated with diluted HRP-labeled anti-human IgG (CUSABIO, Wuhan, China) at 37 °C for 1 h in the dark. After five washes with PBST, the 10% H_2_SO_4_ was used to terminate the chromogenic reaction. Three blanks and five quality controls were set up on each ELISA plate for in-plate quality control and standardization on different plates, respectively.

### Statistical analysis

Data processing from the Huprot™ protein microarray was conducted following the methodology described in our previous study [18]. Optical density (OD) values measured by ELISA were normalized according to the quality control values of each plate. Mann-Whitney U test was used to compare the expression levels of mRNA and TAAbs between the two groups. To establish the cutoff value for each TAAb, we defined it as having a specificity of > 85% and a maximum Youden index (YI). We then calculated diagnostic performance metrics, including sensitivity, specificity, accuracy, YI, positive predictive value (PPV), negative predictive value (NPV), and area under the ROC curve (AUC). Logistic regression was utilized to construct an immunodiagnostic model for the test data. Model robustness was evaluated by an independent validation set and 1000 bootstrap resampling iterations. Differences between the two AUCs were compared using the DeLong test. Data were analyzed using R (version 4.2.3) and SPSS software (version 25).

## Results

### Study design

**Fig. 1:**
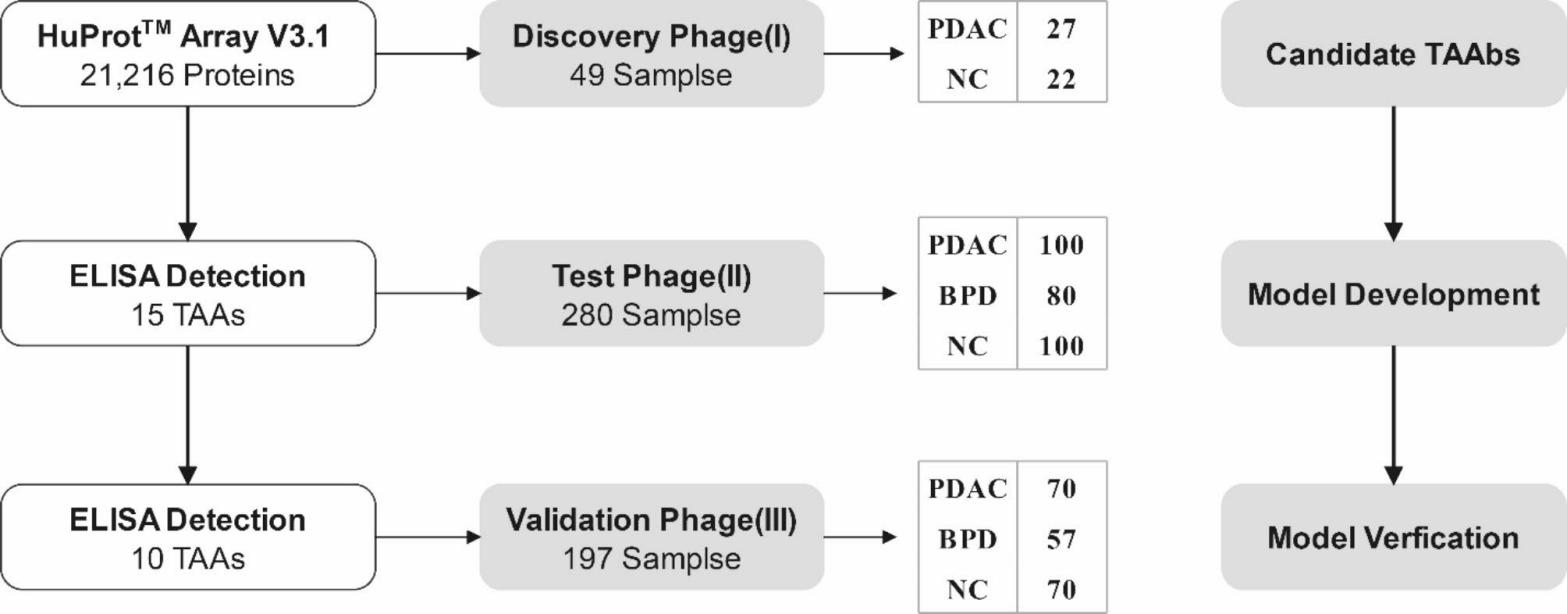
The flow diagram of this study. PDAC: pancreatic ductal adenocarcinoma, NC: normal control, BPD: benign pancreatic diseases

This study included three phases (Fig. 1): discovery phase (I), test phase (II), and validation phase (III). In the discovery phase (I), human autoantibodies profiling was detected using the HuProt™ human proteome chip V3.1 in ten mixed serum PDAC pools and ten NC pools. Thirteen candidate TAAs were identified through bioinformatics analysis, and two TAAs were also included in this study based on our laboratory research [24–26]. In the test phase (II), the assessment of autoantibody levels against 15 candidate TAAs was performed, and a diagnostic model was constructed using 100 PDAC, 80 BPD, and 100 NC samples. Finally, independent verification was carried out using 70 PDAC, 57 BPD, and 70 NC samples in the validation phase (III). The demographic and clinical characteristics of the participants are presented in Table 1.

**Table 1:** Characteristics of participants in discovery, test, and validation phases

	Discovery phase (I)		Test phase (II)			Validation phase (III)		
	PDAC	NC	PDAC	BPD	NC	PDAC	BPD	NC
**N**	27	22	100	80	100	70	57	70
**Gender**								
Male(%)	19(70.37)	13(59.09)	58(58.00)	46(57.50)	58(58.00)	42(60.00)	36(63.16)	42(60.00)
Female(%)	8(29.63)	9(40.91)	42(42.00)	34(42.50)	42(42.00)	28(40.00)	21(36.84)	28(40.00)
**Age, year**								
Mean ± SD	59.67 ± 10.71	54.91 ± 8.90	61.55 ± 11.30	50.16 ± 17.50	61.64 ± 11.32	61.94 ± 10.70	49.16 ± 14.71	61.46 ± 10.90
**CA19-9**								
≤ 37 U/mL			15(15.00)			11(15.71)		
>37 U/mL			59(59.00)			42(60.00)		
Unknown(%)			26(26.00)			17(24.29)		
**TNM**								
I(%)	5(18.52)		24(24.00)			27(38.57)		
II(%)	11(40.74)		12(12.00)			4(5.71)		
III(%)	2(7.41)		7(7.00)			3(4.29)		
IV(%)	9(33.33)		47(47.00)			29(41.43)		
Unknown(%)			10(10.00)			7(10.00)		
**Lymph node Metastasis**								
Yes(%)	5(18.52)		20(20.00)			10(14.29)		
No(%)	17(62.96)		68(68.00)			51(72.86)		
Unknown(%)	5(18.52)		12(12.00)			9(12.86)		
**Distant metastasis**								
Yes(%)	7(25.93)		47(47.00)			28(40.00)		
No(%)	20(74.07)		42(42.00)			33(47.14)		
Unknown(%)			11(11.00)			9(12.86)		

PDAC: pancreatic ductal adenocarcinoma, NC: normal control, BPD: benign pancreatic diseases

### Identification of candidate TAAs based on HuProt^™^ protein microarray

**Fig. 2:**
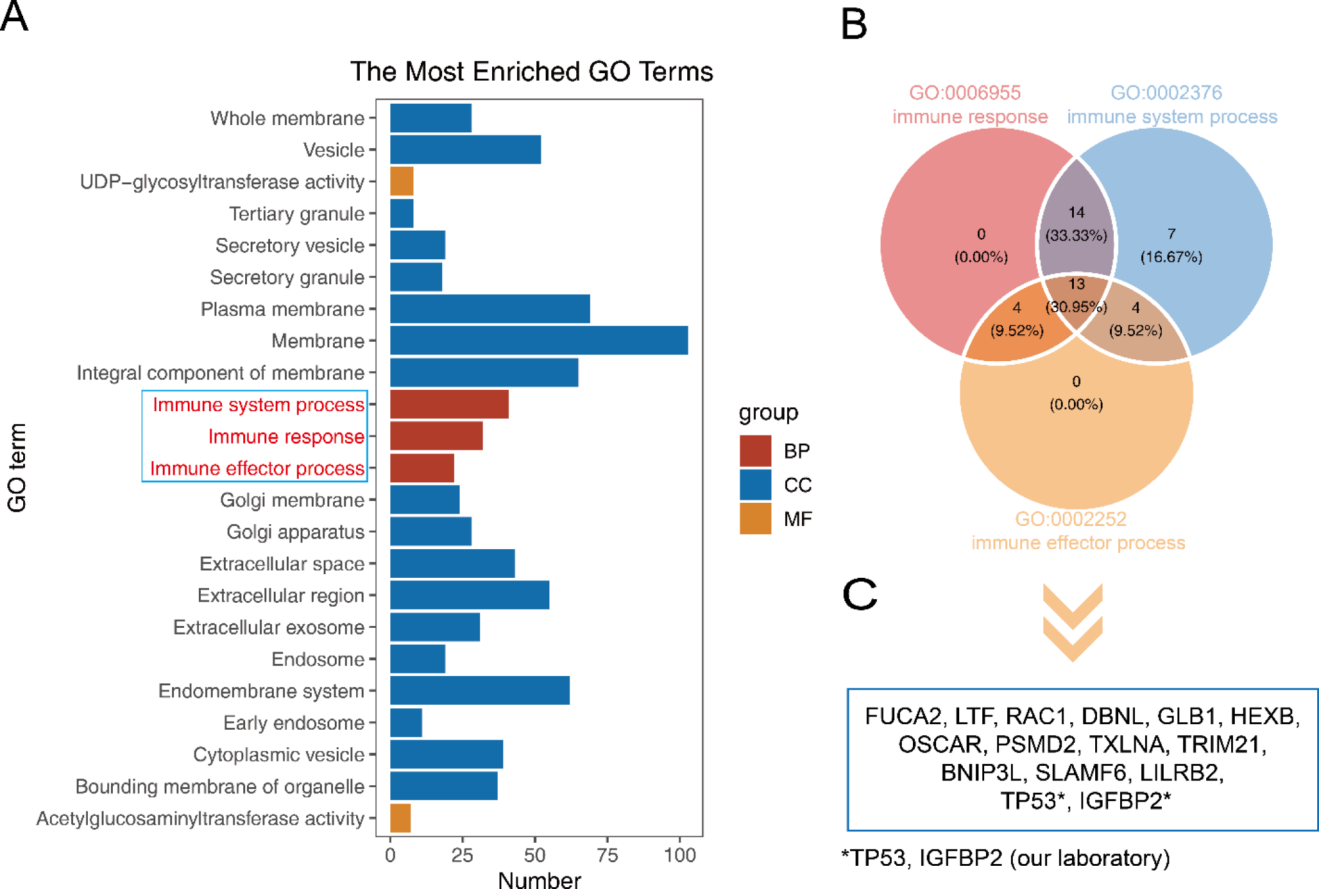
Identification of candidate TAAs based on HuProt™ protein assay. **(A)**. Barplot of GO enrichment analysis results for 167 priority candidate TAAs. **(B)**. Venn Diagram of enrichment results for three terms (GO:0002252, GO:0006955, GO:0002376). **(C)**. Identification of 15 candidate TAAs based on microarray and laboratory data. GO: Gene Ontology, BP: Biological Process, CC: Cellular Component, MF: Molecular Function

**Fig. 3:**
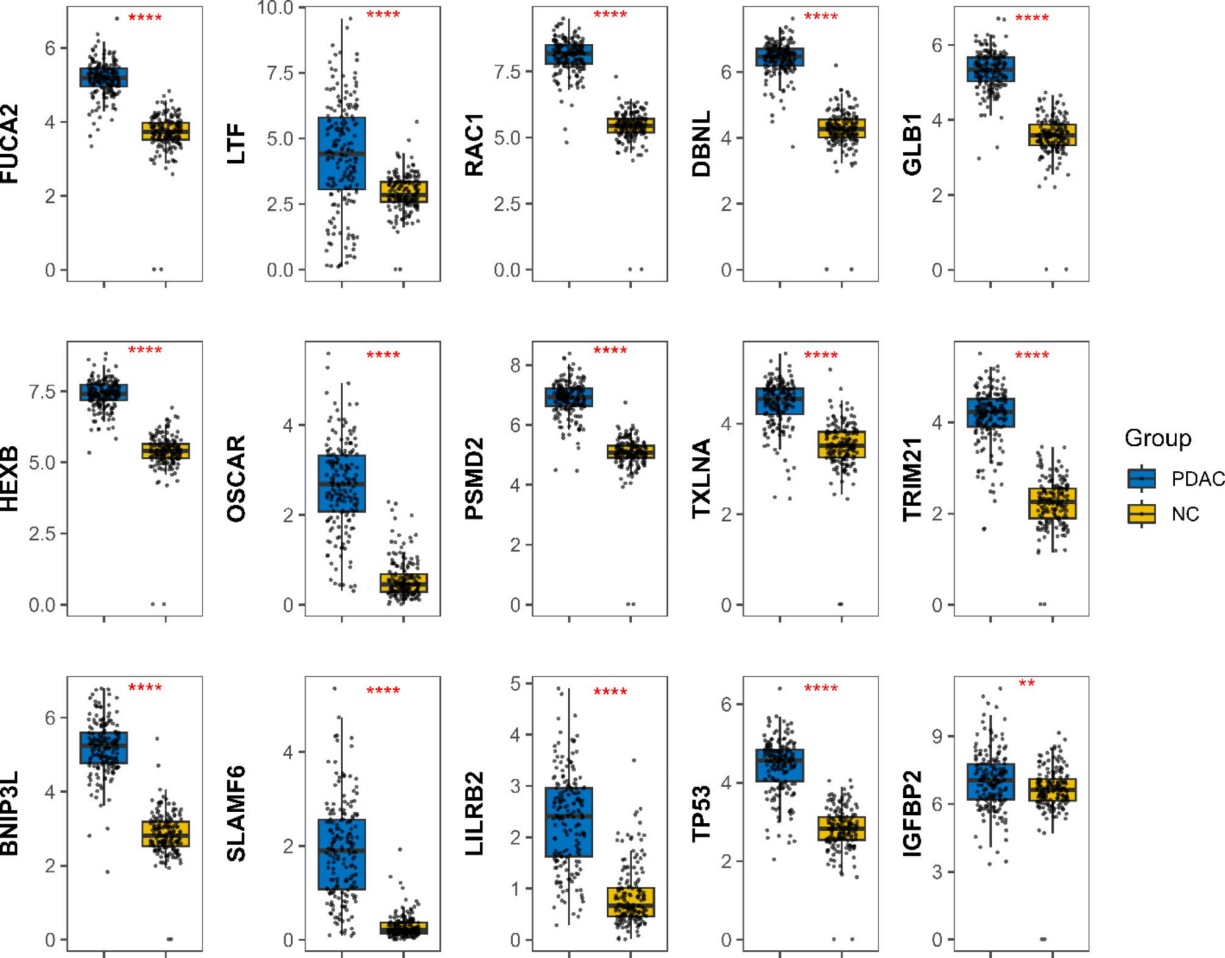
The expression of 15 candidate TAAs at gene expression levels. **: *P* < = 0.01, ****: *P* < = 0.0001. PDAC: pancreatic ductal adenocarcinoma, NC: normal control

In the discovery phase, a total of 167 priority candidate TAAbs were screened based on the criteria (*P* < 0.05, FC > 1.2, PR of PDAC ≥ 50% and NC ≤ 10%). GO enrichment analysis showed that the TAAs corresponding to the TAAbs are closely associated with immune-related biological processes (Fig. 2A). The 13 TAAs were all involved in three biological processes: immune effector process (GO:0002252), immune response (GO:0006955), and immune system process (GO:0002376) (Fig. 2B); p53 (TP53) and p62 (IGFBP2) were included based on previous research. The expression of fifteen TAAs was validated using RNA-Seq data, which showed higher expressions of these genes in tumor samples than in non-tumor samples (Fig. 3). Finally, 15 TAAs (FUCA2, LTF, RAC1, DBNL, GLB1, HEXB, OSCAR, PSMD2, TXLNA, TRIM21, BNIP3L, SLAMF6, LILRB2, TP53, IGFBP2) were selected as candidate autoantigens for subsequent experimental validation to assess autoantibody levels (Fig. 2C). Detailed information about the 15 candidate TAAs is presented in **Table ****S1**.

### Serum levels of autoantibodies in PDAC, NC, and BPD

**Fig. 4:**
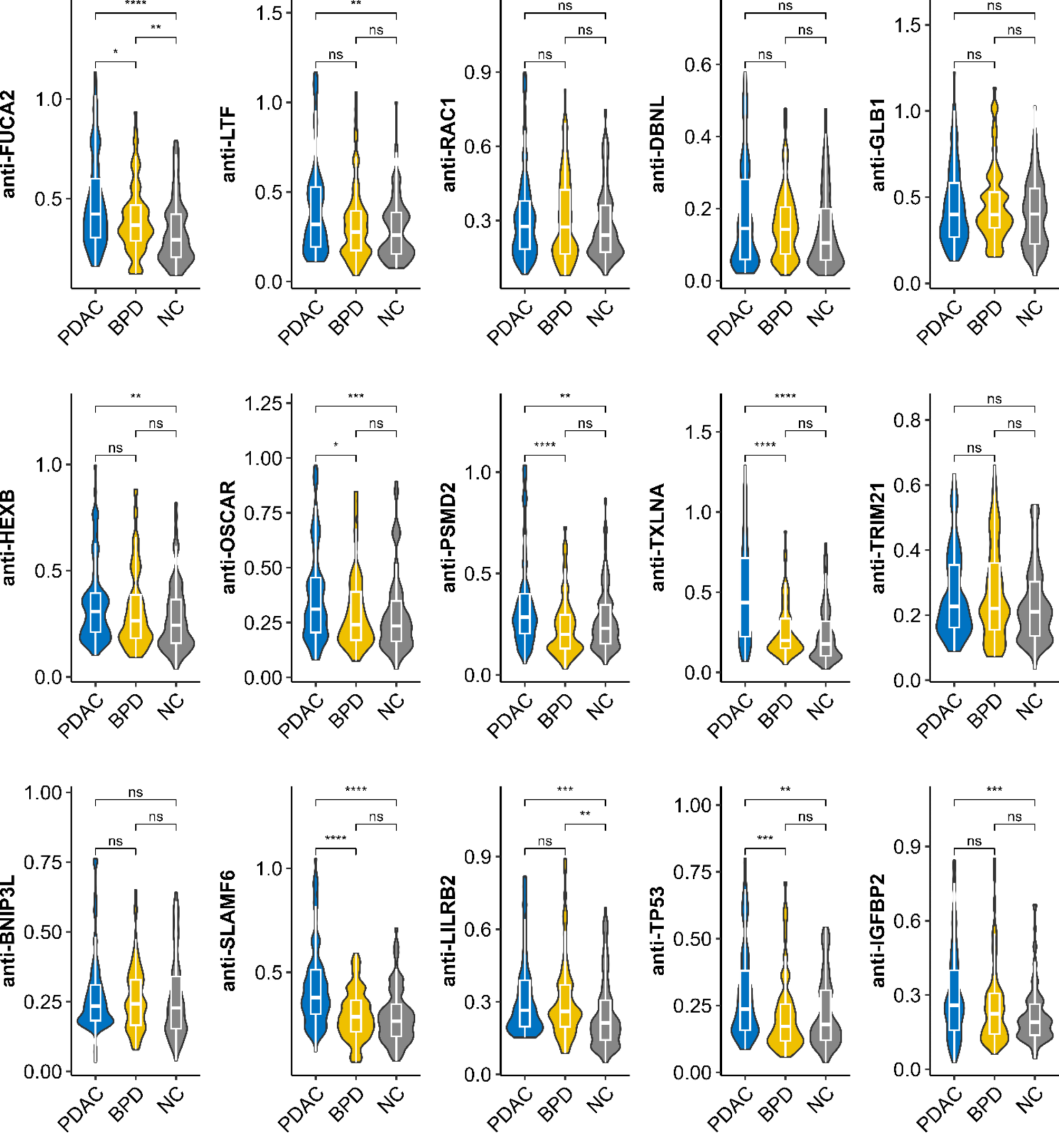
Expression plot of ELISA-detected OD values for 15 TAAbs in the test set. Comparison between PDAC, NC, and BPD samples (ns: *P* > 0.05, *: *P* < = 0.05, **: *P* < = 0.01, ***: *P* < = 0.001, ****: *P* < = 0.0001); PDAC: pancreatic ductal adenocarcinoma, NC: normal control, BPD: benign pancreatic diseases

In the test phase, 280 serum samples (100 PDAC, 80 BPD, and 100 NC) were utilized to assess the levels of autoantibodies against candidate TAAs. The result revealed that 10 TAAbs (anti-FUCA2, anti-LTF, anti-HEXB, anti-OSCAR, anti-PSMD2, anti-TXLNA, anti-SLAMF6, anti-LILRB2, anti-TP53, and anti-IGFBP2) exhibited significant differences between PDAC patients and NC (*P* < 0.05) (Fig. 4). This finding was consistent with the validation phase (**Fig. ****S1**). Furthermore, six TAAbs (anti-FUCA2, anti-OSCAR, anti-PSMD2, anti-TXLNA, anti-SLAMF6, and anti-TP53) demonstrated significantly higher levels in PDAC patients than in BPD (Fig. 4). Additionally, the expression levels of anti-FUCA2 and anti-LILRB2 showed statistically significant differences between the BPD and NC groups (*P* < 0.05) (Fig. 4).

### Diagnostic performance of 10 TAAbs for distinguishing PDAC from NC and BPD groups

**Fig. 5:**
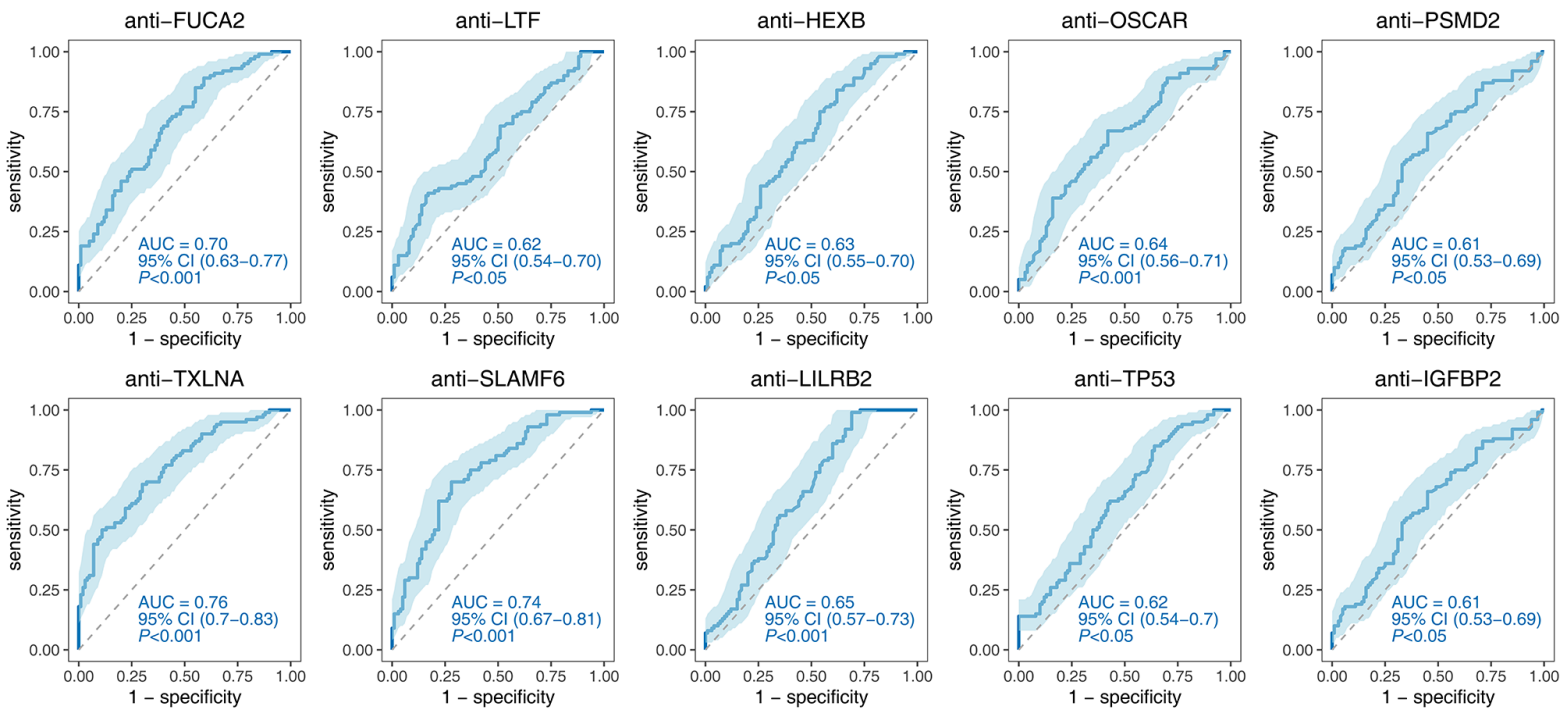
Diagnostic performance of 10 significant TAAbs for distinguishing PDAC from NC

In the test phase, ROC analysis was conducted on the ten significant TAAbs, revealing that compared to the NC group, the diagnostic AUC ranged from 0.61 to 0.76, sensitivity ranged from 7.00 to 50.0%, and specificity ranged from 86.00 to 100.00%. Among them, anti-TXLNA exhibited the highest diagnostic ability with an AUC of 0.76 (95% CI: 0.70–0.83), a sensitivity of 50.00%, and a specificity of 89.00%. Conversely, anti-PSMD2 and anti-IGFBP2 exhibited the lowest diagnostic ability with an AUC of 0.61 (95% CI: 0.53–0.69) for PDAC (Fig. 5; Table 2).

**Table 2:** The diagnostic value of ten TAAbs in the test phase

TAAbs	NC					BPD				
	AUC (95%CI)	Sen (%)	Spe (%)	Acc (%)	*P*	AUC (95%CI)	Sen (%)	Spe (%)	Acc (%)	*P*
FUCA2	0.70(0.63–0.77)	34.00	87.00	60.50	< 0.001	0.60(0.51–0.68)	28.00	86.25	53.89	< 0.05
LTF	0.62(0.54–0.70)	36.00	86.00	61.00	< 0.05	0.58(0.50–0.66)	26.00	85.00	52.22	0.06
HEXB	0.63(0.55–0.70)	19.00	92.00	55.50	< 0.05	0.56(0.47–0.65)	9.00	95.00	47.22	0.164
OSCAR	0.64(0.56–0.71)	29.00	86.00	57.50	< 0.001	0.61(0.53–0.69)	22.00	91.25	52.78	< 0.05
PSMD2	0.61(0.53–0.69)	17.00	95.00	56.00	< 0.05	0.67(0.59–0.75)	26.00	86.25	52.78	< 0.001
TXLNA	0.76(0.70–0.83)	50.00	89.00	69.50	< 0.001	0.73(0.66–0.80)	44.00	92.50	65.56	< 0.001
SLAMF6	0.74(0.67–0.81)	42.00	86.00	64.00	< 0.001	0.72(0.64–0.79)	39.00	88.75	61.11	< 0.001
LILRB2	0.65(0.57–0.73)	7.00	100.00	53.50	< 0.001	0.52(0.43–0.61)	17.00	87.50	48.33	0.652
TP53	0.62(0.54–0.70)	14.00	100.00	57.00	< 0.05	0.66(0.58–0.74)	26.00	90.00	54.44	< 0.001
IGFBP2	0.61(0.53–0.69)	17.00	95.00	56.00	< 0.05	0.67(0.59–0.75)	26.00	86.25	52.78	< 0.001

AUC: area under curve, 95%CI: 95% confidence interval, Sen: sensitivity, Spe: specificity, Acc: Accuracy, NC: normal control, BPD: benign pancreatic diseases

**Fig. 6:**
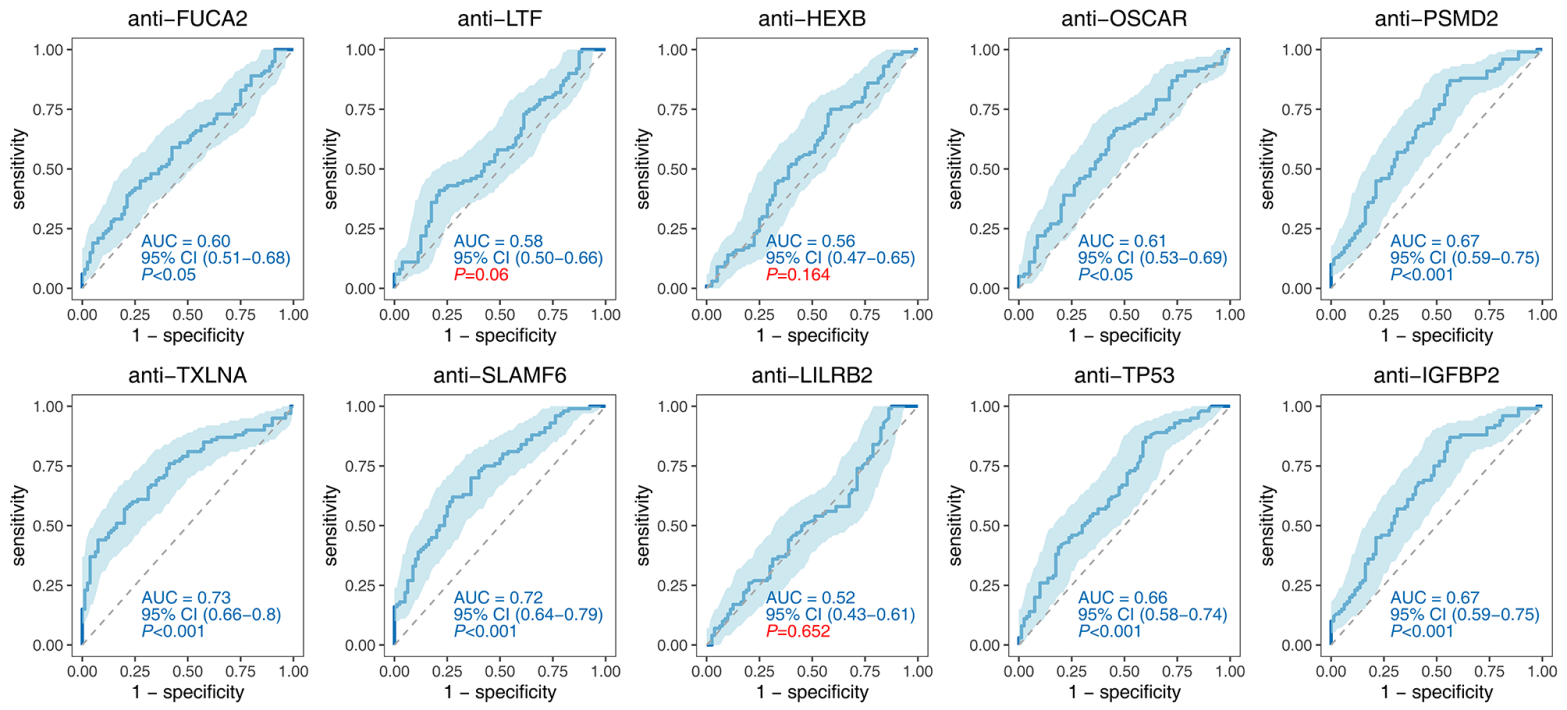
Diagnostic performance of 10 significant TAAbs for distinguishing PDAC from BPD

When using the BPD group as the control, the AUC ranged from 0.52 to 0.73, sensitivity ranged from 9.00 to 44.00%, and specificity ranged from 85.00 to 95.00%. Similarly, anti-TXLNA demonstrated the highest diagnostic ability with an AUC of 0.73 (95% CI: 0.66–0.80), a sensitivity of 44.00%, and a specificity of 92.50%. However, three autoantibodies (anti-LTF, anti-HEXB, and anti-LILRB2) did not exhibit statistical significance (*P* > 0.05) (Fig. 6; Table 2).

### Diagnostic performance of the immunodiagnostic model for distinguishing PDAC from NC

**Fig. 7:**
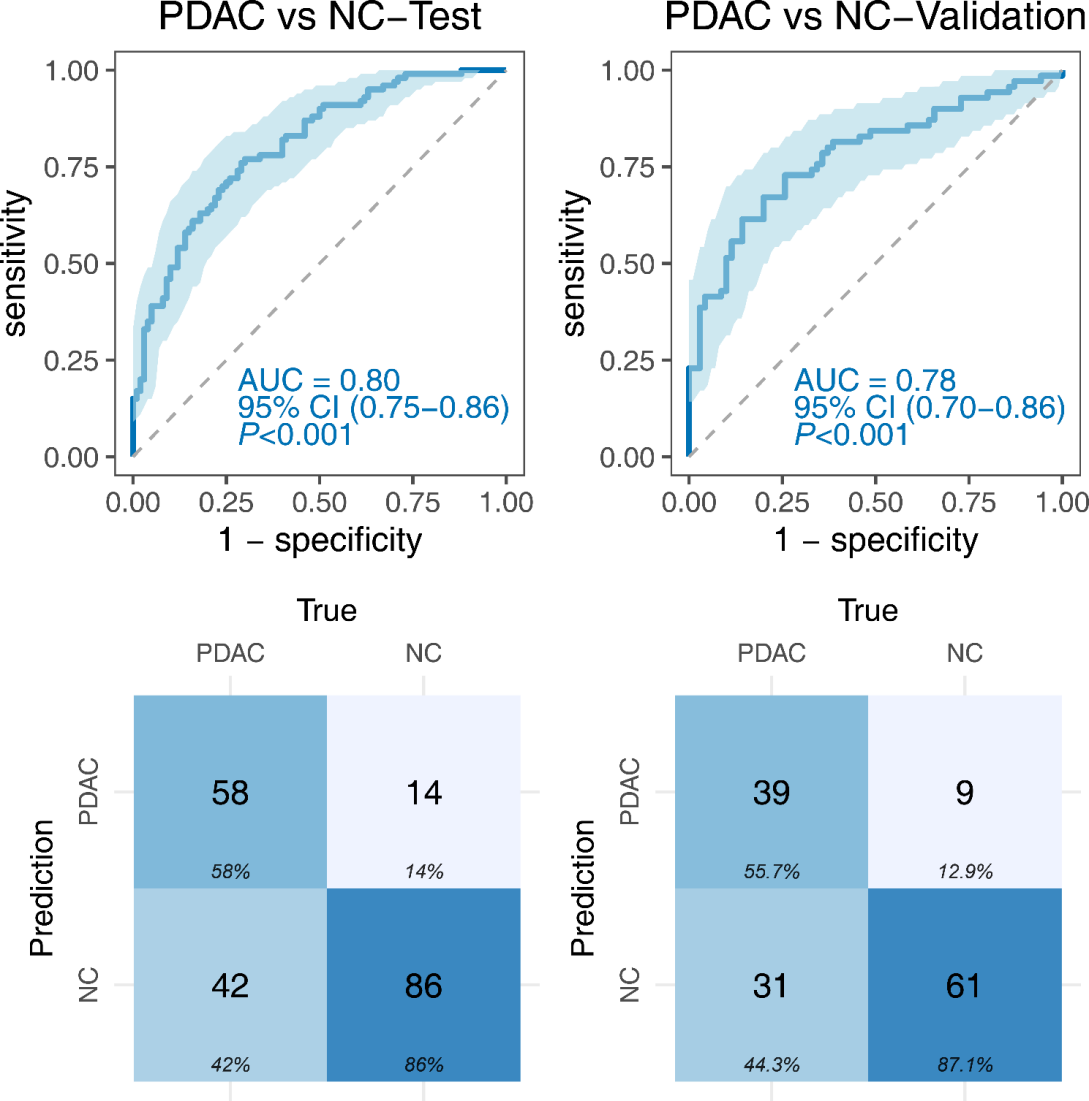
Diagnostic performance of the immunodiagnostic model for distinguishing PDAC from NC

A panel of three TAAbs (anti-HEXB, anti-TXLNA, and anti-SLAMF6) was identified through stepwise logistic regression. The discriminant equation is as follows: PRE (*P*_PDAC_, 3-TAAbs) = 1/(1 + EXP (-(-2.207-2.813×HEXB + 3.671×TXLNA + 5.265×SLAMF6)). The performance evaluation of the established model in the test phase resulted in an AUC of 0.80 (95% CI: 0.75–0.86), with a sensitivity of 58.0% and a specificity of 86.0%. Similarly, in the validation phase, the AUC was 0.78 (95% CI: 0.70–0.86), with a sensitivity of 55.71% and a specificity of 87.14% (Fig. 7; Table 3). The DeLong test demonstrated no significant difference in the discriminatory ability of the model between the training and validation phases (*P* = 0.62). Additionally, the results from 1000 Bootstrap resampling indicated that the mean AUC was 0.80 (95% CI: 0.74–0.86) in the test dataset, 0.79 (95% CI: 0.74–0.84) in the test and validation datasets (**Fig. ****S2**).

**Table 3:** The diagnostic value of immunodiagnostic model

Group	AUC (95%CI)	Sen (%)	Spe (%)	Accuracy	YI	PPV (%)	NPV (%)	*P*
**PDAC vs. NC**								0.62
Test	0.80(0.75–0.86)	58.00	86.00	72.00	0.44	80.56	67.19	
Validation	0.78(0.70–0.86)	58.60	87.14	71.43	0.43	81.25	66.30	
**PDAC vs. BPD**								0.50
Test	0.80(0.73–0.86)	58.00	86.25	70.56	0.43	79.45	66.92	
Validation	0.83(0.76–0.90)	55.71	100.00	75.59	0.56	100.00	64.77	
**CA19-9 status vs. NC**								0.06
CA19-9(+)	0.78(0.72–0.84)	55.45	85.29	74.17	0.41	69.14	76.31	
CA19-9(-)	0.86(0.80–0.93)	65.38	85.29	82.65	0.51	40.48	94.16	
**Early vs. Advanced**								0.23
Early	0.77(0.70–0.84)	54.10	85.29	77.06	0.39	56.90	83.82	
Advanced	0.82(0.77–0.88)	61.11	85.29	76.92	0.46	68.75	80.56	

AUC: area under curve, 95%CI: 95% confidence interval, Sen: sensitivity, Spe: specificity, YI: Youden’s index, PPV: positive predictive value, NPV: negative predictive value, PDAC: pancreatic ductal adenocarcinoma, NC: normal control, BPD: benign pancreatic diseases, *P*: Delong test results between two AUCs.

### Diagnostic performance of the immunodiagnostic model for distinguishing PDAC from BPD

**Fig. 8:**
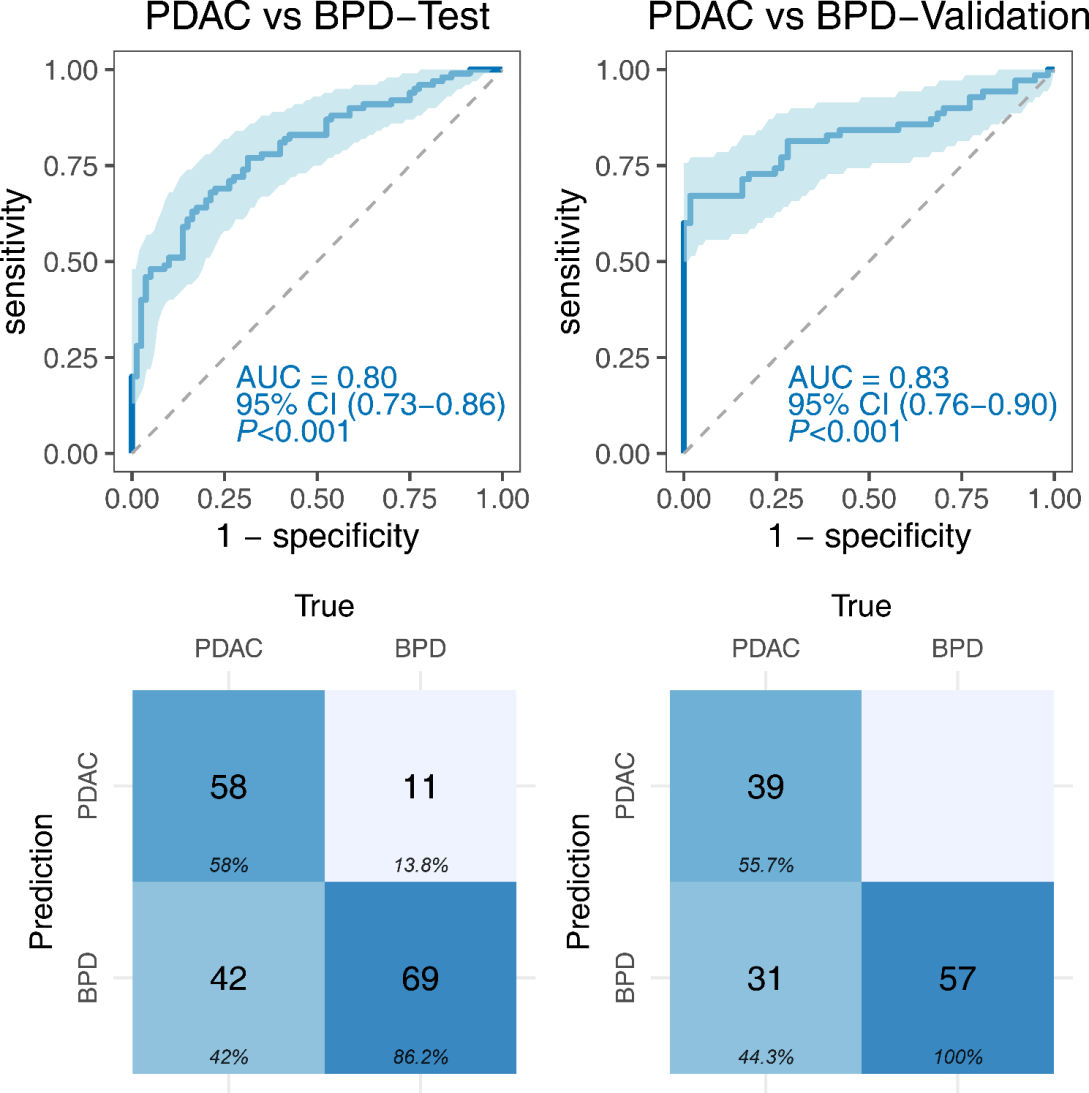
Diagnostic performance of the immunodiagnostic model for distinguishing PDAC from BPD

The developed 3-TAAbs immunodiagnostic model was utilized to evaluate its diagnostic performance in distinguishing PDAC from BPD. It demonstrated an AUC of 0.80 (95% CI: 0.73–0.86), with a sensitivity of 58.00% and a specificity of 86.25% in the test phase. The model achieved an AUC of 0.83 (95% CI: 0.76–0.90) in the validation phase, with a sensitivity of 55.71% and a specificity of 100% (Fig. 8; Table 3).

### Diagnostic performance of the immunodiagnostic model for distinguishing early-stage and advanced-stage PDAC from NC

**Fig. 9:**
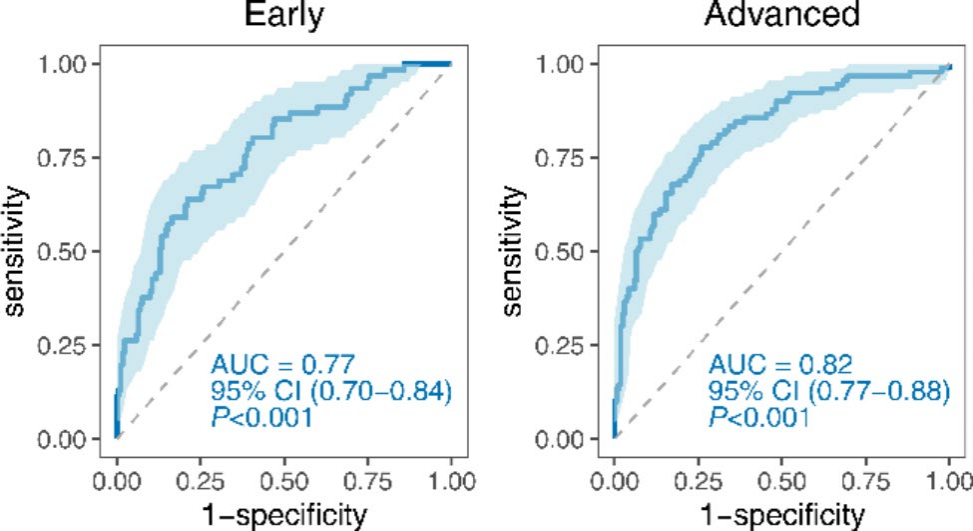
Diagnostic performance of the immunodiagnostic model for distinguishing early-stage and advanced-stage PDAC from NC

Patients with PDAC were categorized into early-stage (I and II) and advanced-stage (III and IV) groups through test and validation data. The immunodiagnostic model demonstrated an AUC of 0.77 (95% CI: 0.70–0.84), with a sensitivity of 54.10% and a specificity of 85.29% for early-stage PDAC. In comparison, for advanced-stage PDAC patients, the immunodiagnostic model revealed an AUC of 0.82 (95% CI: 0.77–0.88), with a sensitivity of 61.11% and a specificity of 85.29% (Fig. 9; Table 3). The DeLong test demonstrated no statistically significant difference (*P* = 0.23) (Table 3).

### Enhanced diagnostic performance of the three-TAAbs immunodiagnostic model combined with CA19-9

**Fig. 10:**
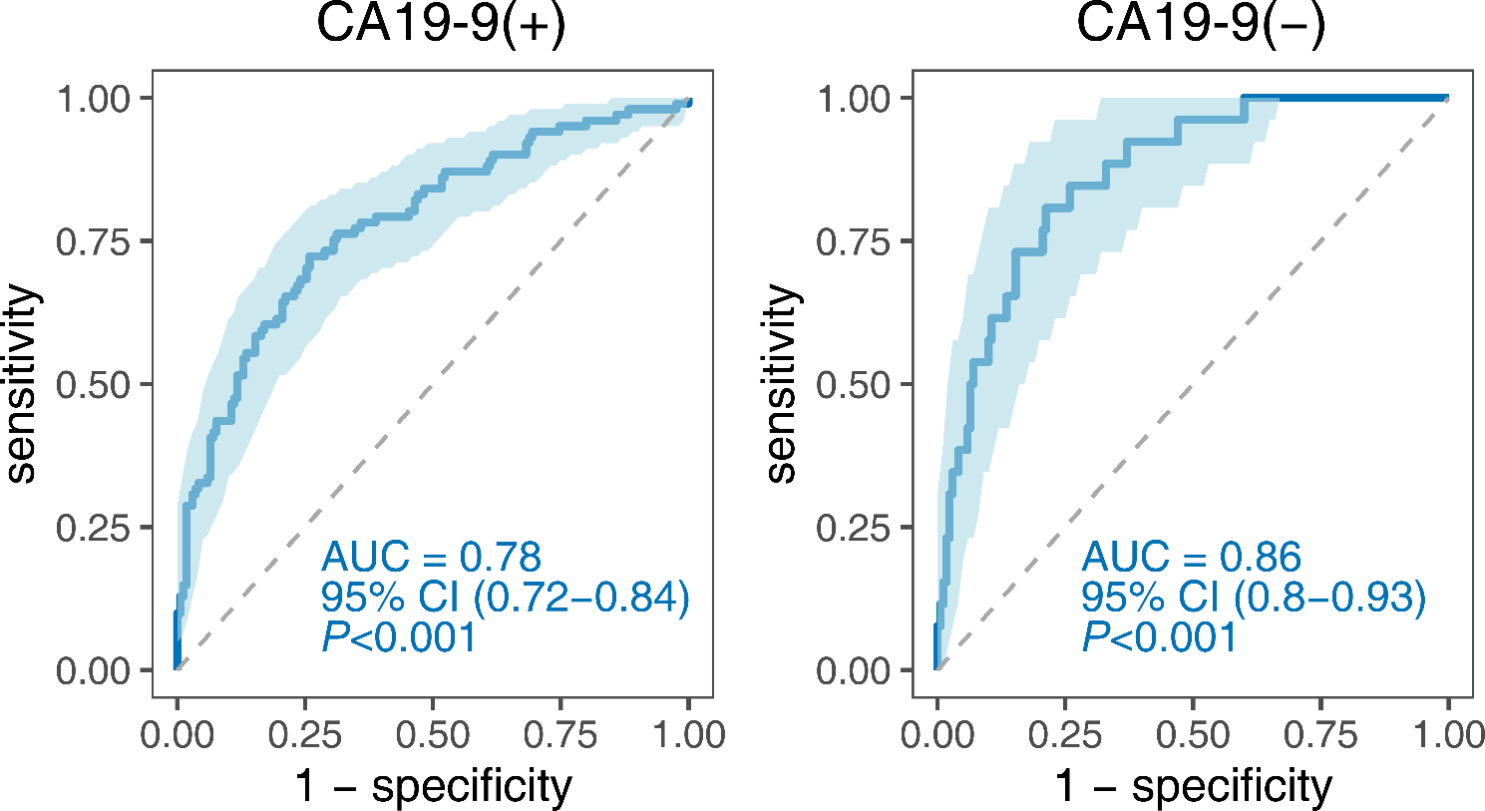
Immunodiagnostic model performance for distinguishing PDAC from CA19-9-positive and CA19-9-negative groups

Using a cutoff value of CA19-9 = 37 U/mL, the PDAC patients (n = 127) were divided into CA19-9-positive and CA19-9-negative groups. Among the CA19-9-positive PDAC cases, the immunodiagnostic model exhibited an AUC of 0.78 (95% CI: 0.72–0.84), with a sensitivity of 55.45% and a specificity of 85.29%. Conversely, for CA19-9-negative PDAC diagnosis, the AUC was 0.86 (95% CI: 0.80–0.93), with a sensitivity of 65.38% and a specificity of 85.29% (Fig. 10; Table 3). When the CA19-9 and the immunodiagnostic model were combined in parallel, the PR increased to 92.91%, surpassing the PR of the model and CA19-9 alone (57.48% and 79.52%, respectively). This difference was statistically significant (*P* < 0.05) (Table 4, **Fig. ****S3**).

**Table 4:** Comparison of single and parallel detection of CA19-9 and model

Methods	Number of positive	Number of negative	Positive rate
CA19-9	101	26	79.52^a^
3-TAAbs Panel	73	54	57.48^b^
Panel + CA19-9	118	9	92.91^c^

^a^: The chi-square test showed *P* < 0.001 for CA19-9 and 3-TAAbs Panel; ^b^: The chi-square test showed *P* < 0.001 for CA19-9 and Panel + CA19-9; ^c^: The chi-square test showed *P* < 0.001 for 3-TAAbs Panel and Panel + CA19-9.

## Discussion

Pancreatic ductal adenocarcinoma is characterized by its highly malignant nature and poor prognosis, presenting a significant clinical challenge regarding accurate diagnosis [5]. Serum TAAbs have emerged as promising biomarkers for cancer diagnosis [6]. This study employed high-throughput protein assays to identify ten novel TAAbs that exhibited differential expression between PDAC and normal samples. A three-TAAbs immunodiagnostic model was also developed, demonstrating robust diagnostic performance. Notably, when combined with the commonly used biomarker CA19-9, this model exhibited superior performance in detecting PDAC.

The human protein microarray offers a platform for high-throughput, comprehensive, and rapid analysis, making it advantageous for identifying TAAbs and significant for early tumor diagnosis [17–21]. Zhuang et al. have utilized the approach to identify five TAAbs in pancreatic cancer: anti-CLDN17, anti-KCNN3, anti-SLAMF7, anti-SLC22A11, and anti-OR51F2 [27]. In our study, we employed protein microarray in pooled samples to screen for candidate TAAbs in a cost-effective and time-efficient manner. Considering the close connection between autoantibody production and immune response mechanisms [7, 9, 28], we mainly focused on immune response-related TAAs as potential biomarkers for further validation. Additionally, this study included the two promising TAAs (p53/TP53 and p62/IGFBP2) previously identified in our laboratory [24–26]. Moreover, we performed bioinformatics analysis to strengthen the robustness of the selected TAAs based on the protein microarray results.

Among the identified TAAs, previous studies have reported associations between FUCA2 [29], LTF [30], OSCAR [31], PSMD2 [32–35], LILRB2 [36], SLAMF6 [37], TP53 [38], IGFBP2 [39] and immune infiltration as well as the immunosuppressive microenvironment. Additionally, it has been documented that LTF [40], TXLNA [41], LILRB2 [42], SLAMF6 [43], TP53 [38], IGFBP2 [39] are associated with the development of pancreatic cancer. To the best of our knowledge, only TP53 and IGFBP2 have been investigated for their diagnostic potential as autoantibodies in pancreatic cancer [44, 45], while the diagnostic application of TAAbs targeting other identified TAAs has not been reported in the literature to date.

Based on ELISA results, we focused on ten significant TAAbs (anti-FUCA2, anti-LTF, anti-HEXB, anti-OSCAR, anti-PSMD2, anti-TXLNA, anti-LILRB2, anti-SLAMF6, anti-TP53, and anti-IGFBP2) as candidate biomarkers for PDAC diagnosis. These TAAbs exhibited good sensitivity and specificity, with an AUC range from 0.61 to 0.76, a sensitivity range from 7.00 to 50.00%, and a specificity range from 86.00 to 100.00%. Seven out of the ten TAAbs showed the ability to distinguish PDAC from BPD. Remarkably, anti-TXLNA demonstrated exceptional performance in distinguishing PDAC from NC (AUC = 0.76) and BPD (AUC = 0.73). Our findings differ from a previous study by Zhuang et al. [27], which utilized the same protein microarray. Several factors may contribute to these discrepancies, including variations in study populations, sample sizes, clinical characteristics, serum storage conditions, and immune profiles. Furthermore, it is noteworthy that we observed dual diagnostic values of anti-SLAMF6 in our study, with an AUC of 0.74 for PDAC and 0.72 for BPD. Interestingly, the autoantibody against SLAMF7, another member of the SLAM family, has been reported to have an AUC of 0.79 in pancreatic cancer according to the study by Zhuang et al. [27].

The combination of biomarker signatures has shown promise in enhancing the accuracy of cancer diagnosis [46]. A recent study reported an excellent AUC of 0.925 by combining 29 TAAbs in an immunodiagnostic model [47]. Our study utilized logistic regression analysis to identify an optimized panel of three TAAbs (anti-HEXB, anti-TXLNA, and anti-SLAMF6). Two methods validated the immunodiagnostic model, and the results confirmed its robust discriminatory ability and valuable performance with an AUC of 0.78 (95% CI: 0.70–0.86). Notably, this model demonstrated effective differentiation between PDAC and BPD patients, with an AUC of 0.83 (95% CI: 0.76–0.90). However, it should be mentioned that the performance of the model in early-stage PDAC was limited, with an AUC of 0.77 (95% CI: 0.70–0.84). This limitation could be attributed to the relatively small sample size of early-stage PDAC cases in our study.

CA19-9 is the most commonly used biomarker for diagnosing pancreatic cancer, but its limited specificity hinders diagnostic accuracy [15]. Notably, TAAbs often possess sufficient specificity, and combining a TAAb panel with CA19-9 may enhance diagnostic efficiency for pancreatic cancer. Zhuang et al. discovered that combining anti-CLDN17 with CA19-9 resulted in superior diagnostic performance, with an AUC of 0.93 [27]. Another study also found that combining CTC with CA19-9 improved the diagnostic accuracy of pancreatic cancer, achieving an AUC of 0.95 [48]. In our study, we found that the developed model using three TAAbs achieved an AUC of 0.86 for patients who were CA19-9 negative, suggesting that our model could enhance diagnostic ability when combined with CA19-9. Therefore, we combined CA19-9 with the developed model, resulting in an increased positive rate to 92.91%. It demonstrates that the 3-TAAbs immunodiagnostic model plays a valuable complementary role for CA19-9.

These findings suggest that the developed model holds promise as a novel tool for the clinical diagnosis of pancreatic cancer. The use of high-throughput proteome microarray technology, along with a three-stage strategy, enabled the efficient identification of TAAbs with diagnostic potential. Additionally, rigorous design and methodology were implemented to ensure the reproducibility and authenticity of the study results, including sample inclusion criteria and ELISA detection. However, certain limitations should be acknowledged. Firstly, our study only included PDAC specimens from three hospitals with limited sample sizes, and further evaluation of the immunodiagnostic model in larger sample populations is warranted to determine its diagnostic value for PDAC, particularly in early-stage cases. Secondly, the study focused specifically on immune-related TAAs, potentially missing out on other TAAs of diagnostic value. It would be valuable to consider the inclusion of additional TAAs in future studies to broaden the scope of potential diagnostic biomarkers.

## Conclusion

In conclusion, ten potential TAAbs were identified for the PDAC diagnosis. We also developed a robust diagnostic model that accurately distinguishes PDAC from healthy individuals and patients with benign pancreatic diseases. Importantly, this model demonstrated superior diagnostic efficiency by combining CA19-9. These findings highlight the promising potential of our approach as a non-invasive tool for the diagnosis of pancreatic cancer.

### Electronic supplementary material

Below is the link to the electronic supplementary material.

Supplementary Material 1: Fig. S1. The expression plot of OD values of ELISA for 10 TAAbs in the validation set. ns: *P* >0.05, *: *P*<=0.05, **: *P*<=0.01, ***: *P*<=0.001, ****: *P*<=0.0001; PDAC: pancreatic ductal adenocarcinoma, BPD: benign pancreatic diseases, NC: normal control.

Supplementary Material 2: Fig. S2. ROC curve of validation for the 3-TAAbs model using 1000 bootstrap resampling.

Supplementary Material 3: Fig. S3. Barplot of positive rate for single and parallel detection of CA19-9 and model.

Supplementary Material 4: Table S1. The descriptions of the 15 candidate TAAs.

## Data Availability

All the data in this study are available from the corresponding authors upon a reasonable requirement.
