# Supplementary figures and images for: A novel autoantibody signatures for enhanced clinical diagnosis of pancreatic ductal adenocarcinoma
Source: Cancer Cell Int. 2023 Nov 16;23:273. doi: 10.1186/s12935-023-03107-1 (PMC10655307; doi:10.1186/s12935-023-03107-1)

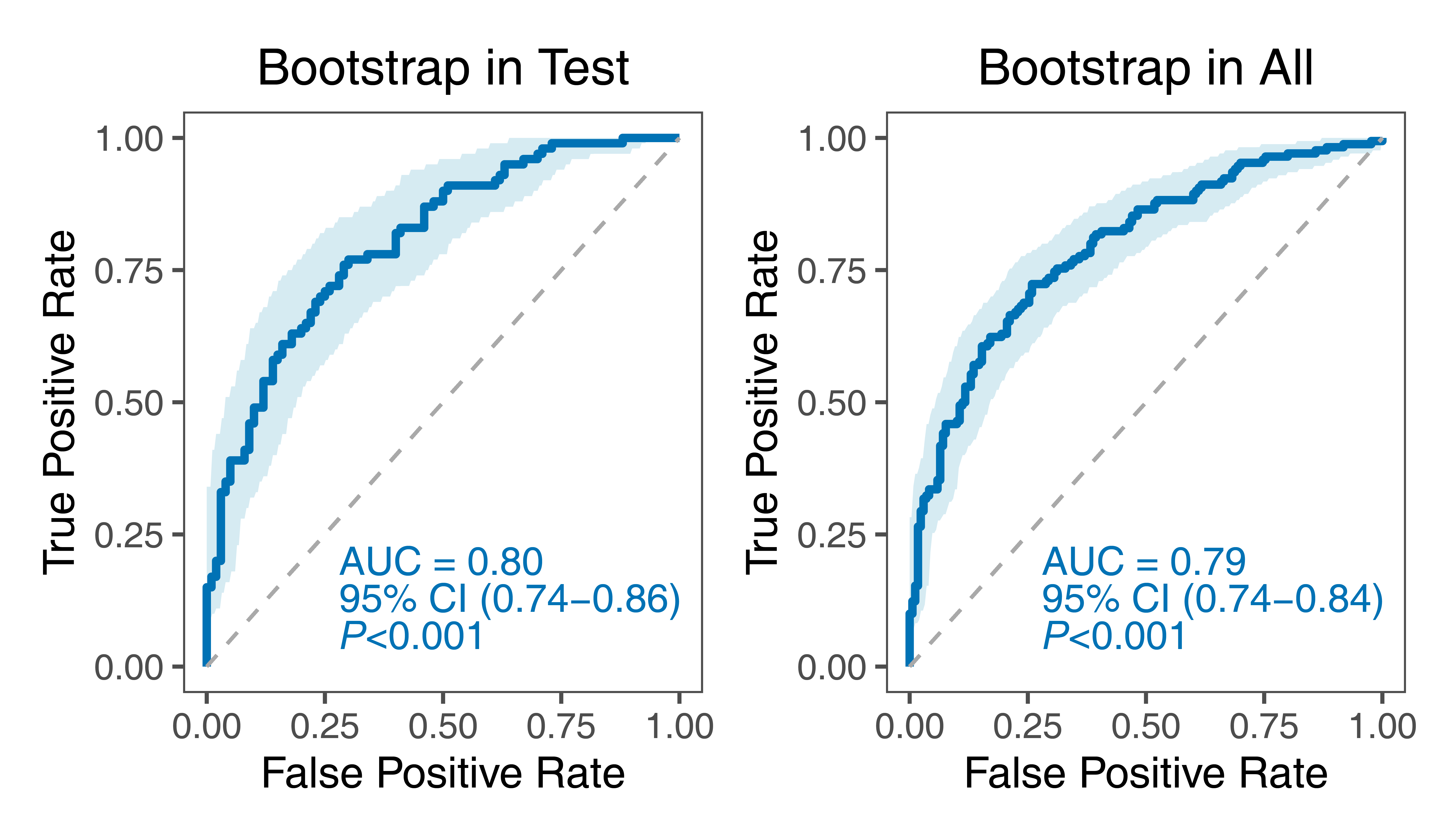

Supplement: Supplementary file 2 — Supplementary Material 2: Fig. S2. ROC curve of validation for the 3-TAAbs model using 1000 bootstrap resampling. [file 12935_2023_3107_MOESM2_ESM.png]

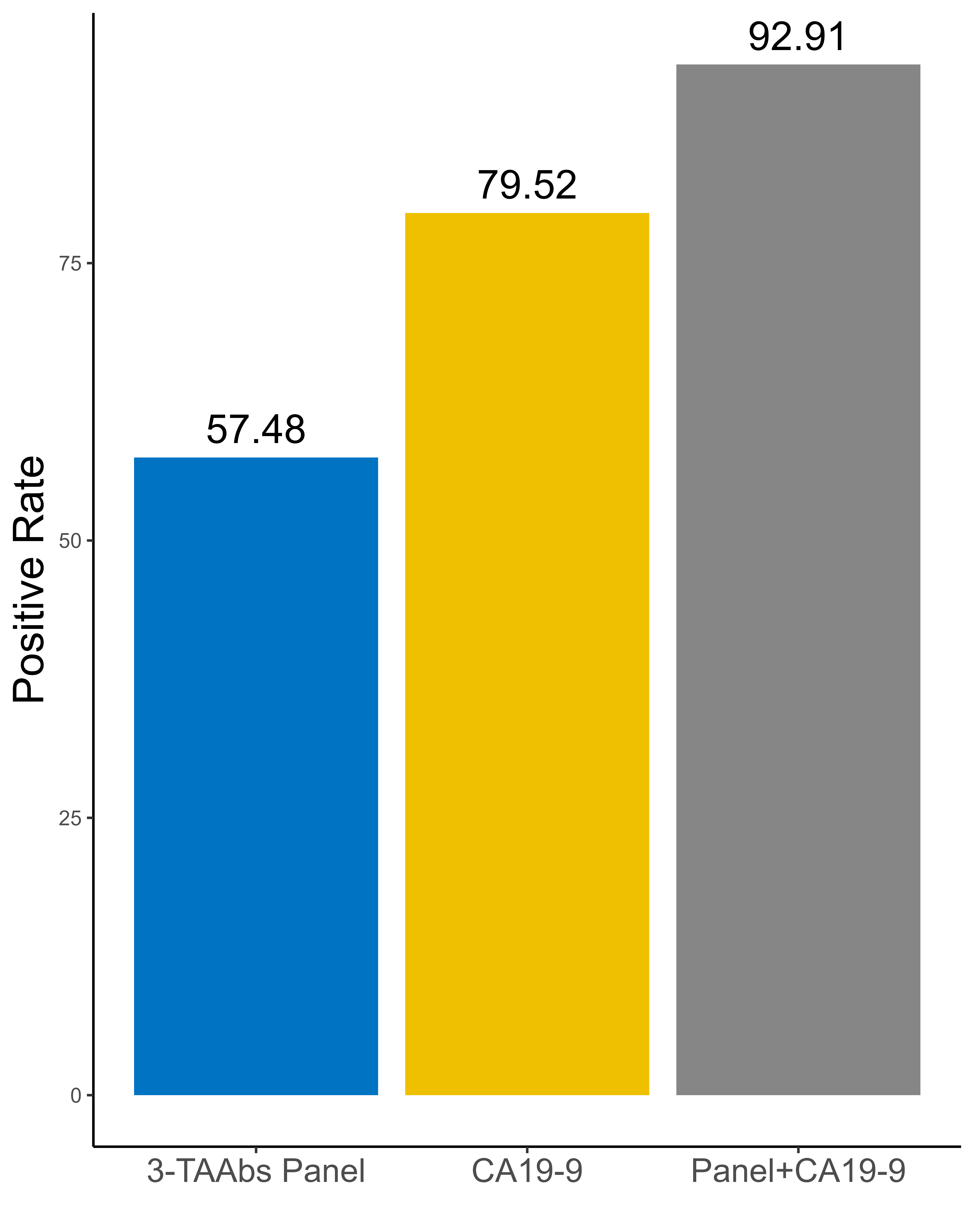

Supplement: Supplementary file 3 — Supplementary Material 3: Fig. S3. Barplot of positive rate for single and parallel detection of CA19-9 and model. [file 12935_2023_3107_MOESM3_ESM.png]
