# Supplementary material for: A novel autoantibody signatures for enhanced clinical diagnosis of pancreatic ductal adenocarcinoma
Source: Cancer Cell Int. 2023 Nov 16;23:273. doi: 10.1186/s12935-023-03107-1 (PMC10655307; doi:10.1186/s12935-023-03107-1)
Supplement: Supplementary file 4 — Supplementary Material 4: Table S1. The descriptions of the 15 candidate TAAs. [file 12935_2023_3107_MOESM4_ESM.docx]

**Table S1 The descriptions of the 15 candidate TAAs.**

| **TAAs** | **Full Name** | **Function/process** | **Literature** |
| --- | --- | --- | --- |
| FUCA2 | Alpha-L-Fucosidase 2 | enables alpha-L-fucosidase activity; enables protein binding; involved_in fucose metabolic process; involved_in glycoside catabolic process | immunosuppressive microenvironment[1]; promote cancer growth and metastasis[2,3]; |
| LTF | Lactotransferrin | regulation of iron homeostasis, host defense against a broad range of microbial infections, anti-inflammatory activity, regulation of cellular growth and differentiation and protection against cancer development and metastasis | regulates the immune microenvironment[4]; pancreatic cancer development[5] |
| RAC1 | Rac Family Small GTPase 1 | enables G protein activity, enables enzyme binding | promote tumor progression[6] |
| DBNL | Drebrin Like | involved in adaptive immune response, enables enzyme activator activity. | antiproliferative effects[7] |
| GLB1 | Galactosidase Beta 1 | enables protein binding. | senescence-like phenotype[8]. |
| HEXB | Hexosaminidase Subunit Beta | enables N-acetyl-beta-D-galactosaminidase activity; enables beta-N-acetylhexosaminidase activity; enables protein binding | poor prognosis in cancer patients[9,10]; |
| OSCAR | Osteoclast Associated Ig-Like Receptor | innate and adaptive immune responses; enables collagen receptor activity | promote proliferation and migration of lung adenocarcinoma[11]; proinflammatory[12] |
| PSMD2 | Proteasome 26S Subunit Ubiquitin Receptor, Non-ATPase 2 | processing of class I MHC peptides; enables enzyme regulator activity; enables protein binding; involved in regulation of protein catabolic process | immune infiltration[13–16]; |
| TXLNA | Taxilin Alpha | B cell activation; enables protein binding; enables syntaxin binding | prognosis and targets for pancreatic cancer[17]; metastatic and invasive[18] |
| TRIM21 | Tripartite Motif Containing 21 | involved in innate immune response, involved in negative regulation of innate immune response. | cancer metabolism, immunity, especially the immune response to inflammation[19,20] |
| BNIP3L | BCL2 Interacting Protein 3 Like | enables protein binding, involved in defense response to virus | induces mitophagy[21,22] |
| SLAMF6 | SLAM Family Member 6 | involved in T cell activation; enables protein binding; involved in innate immune response | immune regulation[23]; prognostic signature of pancreatic cancer[24]; |
| LILRB2 | Leukocyte Immunoglobulin Like Receptor B2 | enables MHC class I protein binding; enables inhibitory MHC class I receptor activity; involved in Fc receptor mediated inhibitory signaling pathway | epithelial-to-mesenchymal transition[25]; reprograms tumor-associated myeloid cells[26] |
| p53/TP53 | Tumor protein p53 | programmed cell death; DNA repair; oncogene induced senescence; involved in B cell lineage commitment | immune evasion[27]; |
| p62/IGFBP2 | Insulin Like Growth Factor Binding Protein 2 | enables insulin-like growth factor I binding; involved in positive regulation of activated T cell proliferation; involved in cellular response to hormone stimulus; | promoted an immunosuppressive microenvironment and tumor growth[28]; |

**Reference**

[1] A. Zhong, T. Chen, Y. Xing, X. Pan, M. Shi, FUCA2 Is a Prognostic Biomarker and Correlated With an Immunosuppressive Microenvironment in Pan-Cancer, Front. Immunol. 12 (2021) 758648. https://doi.org/10.3389/fimmu.2021.758648.

[2] Q. Liu, H.-T. Dong, T. Zhao, F. Yao, Y. Xu, B. Chen, Y. Wu, F. Jin, P. Xing, Cancer-associated adipocytes release FUCA2 to promote aggressiveness in TNBC, Endocr. Relat. Cancer. 29 (2022) 139–149. https://doi.org/10.1530/ERC-21-0243.

[3] M.W. Leal Quirino, A.P. de B. Albuquerque, M. de F.D. de Souza, A.F. da S. Filho, M.R. Martins, M.G. da R. Pitta, M.C. Pereira, M.J.B. de Melo, FUCA2 and TSTA3 expression in gastric cancer: candidate biomarkers of malignant transformation, Folia Histochem. Cytobiol. 60 (2022) 335–343. https://doi.org/10.5603/FHC.a2022.0031.

[4] Q. Zhao, Y. Cheng, Y. Xiong, LTF Regulates the Immune Microenvironment of Prostate Cancer Through JAK/STAT3 Pathway, Front. Oncol. 11 (2021) 692117. https://doi.org/10.3389/fonc.2021.692117.

[5] T. Takata, Y. Ishigaki, T. Shimasaki, H. Tsuchida, Y. Motoo, A. Hayashi, N. Tomosugi, Characterization of proteins secreted by pancreatic cancer cells with anticancer drug treatment in vitro, Oncol. Rep. 28 (2012) 1968–1976. https://doi.org/10.3892/or.2012.2020.

[6] Z. Li, S. Cao, Y. Sun, Z. Niu, X. Liu, J. Niu, Y. Zhou, TIPE3 is a candidate prognostic biomarker promoting tumor progression via elevating RAC1 in pancreatic cancer, Mol. Cancer. 21 (2022) 160. https://doi.org/10.1186/s12943-022-01626-5.

[7] D. Treue, M. Bockmayr, A. Stenzinger, D. Heim, S. Hester, F. Klauschen, Proteogenomic systems analysis identifies targeted therapy resistance mechanisms in EGFR-mutated lung cancer, Int. J. Cancer. 144 (2019) 545–557. https://doi.org/10.1002/ijc.31845.

[8] J. Wagner, N. Damaschke, B. Yang, M. Truong, C. Guenther, J. McCormick, W. Huang, D. Jarrard, Overexpression of the novel senescence marker β-galactosidase (GLB1) in prostate cancer predicts reduced PSA recurrence, PloS One. 10 (2015) e0124366. https://doi.org/10.1371/journal.pone.0124366.

[9] Y. Zhao, J. Zhang, S. Wang, Q. Jiang, K. Xu, Identification and Validation of a Nine-Gene Amino Acid Metabolism-Related Risk Signature in HCC, Front. Cell Dev. Biol. 9 (2021) 731790. https://doi.org/10.3389/fcell.2021.731790.

[10] M. Jia, W. Zhang, J. Zhu, C. Huang, J. Zhou, J. Lian, Y. Wang, H. Teng, Z. Huang, Microglia-Specific Expression of HEXA and HEXB Leads to Poor Prognosis in Glioblastoma Patients, Front. Oncol. 11 (2021) 685893. https://doi.org/10.3389/fonc.2021.685893.

[11] D. Wang, N. Yang, S. Xie, Sex-biased ceRNA networks reveal that OSCAR can promote proliferation and migration of lung adenocarcinoma in women, Clin. Exp. Pharmacol. Physiol. 47 (2020) 1350–1359. https://doi.org/10.1111/1440-1681.13318.

[12] H.S. Schultz, L. Guo, P. Keller, A.J. Fleetwood, M. Sun, W. Guo, C. Ma, J.A. Hamilton, O. Bjørkdahl, M.W. Berchtold, S. Panina, OSCAR-collagen signaling in monocytes plays a proinflammatory role and may contribute to the pathogenesis of rheumatoid arthritis, Eur. J. Immunol. 46 (2016) 952–963. https://doi.org/10.1002/eji.201545986.

[13] S. Wang, H. Wang, S. Zhu, Z. Wang, PSMD2 promotes the progression of bladder cancer and is correlated with immune infiltration, Front. Oncol. 12 (2022) 1058506. https://doi.org/10.3389/fonc.2022.1058506.

[14] H. Zhao, G. Lu, Prognostic Implication and Immunological Role of PSMD2 in Lung Adenocarcinoma, Front. Genet. 13 (2022) 905581. https://doi.org/10.3389/fgene.2022.905581.

[15] D.T.M. Xuan, C.-C. Wu, T.-J. Kao, H.D.K. Ta, G. Anuraga, V. Andriani, M. Athoillah, C.-C. Chiao, Y.-F. Wu, K.-H. Lee, C.-Y. Wang, J.-Y. Chuang, Prognostic and immune infiltration signatures of proteasome 26S subunit, non-ATPase (PSMD) family genes in breast cancer patients, Aging. 13 (2021) 24882–24913. https://doi.org/10.18632/aging.203722.

[16] B. Shen, G. Zhang, Y. Liu, J. Wang, J. Jiang, Identification and Analysis of Immune-Related Gene Signature in Hepatocellular Carcinoma, Genes. 13 (2022) 1834. https://doi.org/10.3390/genes13101834.

[17] S. Lv, G. Zhang, L. Xie, Z. Yan, Q. Wang, Y. Li, L. Zhang, Y. Han, H. Li, Y. Du, Y. Yang, X. Guo, High TXLNA Expression Predicts Favourable Outcome for Pancreatic Adenocarcinoma Patients, BioMed Res. Int. 2020 (2020) 2585862. https://doi.org/10.1155/2020/2585862.

[18] T. Mashidori, H. Shirataki, T. Kamai, F. Nakamura, K.-I. Yoshida, Increased alpha-taxilin protein expression is associated with the metastatic and invasive potential of renal cell cancer, Biomed. Res. Tokyo Jpn. 32 (2011) 103–110. https://doi.org/10.2220/biomedres.32.103.

[19] Q. Zhang, L. Lv, P. Ma, Y. Zhang, J. Deng, Y. Zhang, Identification of an Autophagy-Related Pair Signature for Predicting Prognoses and Immune Activity in Pancreatic Adenocarcinoma, Front. Immunol. 12 (2021) 743938. https://doi.org/10.3389/fimmu.2021.743938.

[20] X. Chen, M. Cao, P. Wang, S. Chu, M. Li, P. Hou, J. Zheng, Z. Li, J. Bai, The emerging roles of TRIM21 in coordinating cancer metabolism, immunity and cancer treatment, Front. Immunol. 13 (2022) 968755. https://doi.org/10.3389/fimmu.2022.968755.

[21] Y. Li, W. Zheng, Y. Lu, Y. Zheng, L. Pan, X. Wu, Y. Yuan, Z. Shen, S. Ma, X. Zhang, J. Wu, Z. Chen, X. Zhang, BNIP3L/NIX-mediated mitophagy: molecular mechanisms and implications for human disease, Cell Death Dis. 13 (2021) 14. https://doi.org/10.1038/s41419-021-04469-y.

[22] Y.-Y. Chen, W.-H. Wang, L. Che, Y. Lan, L.-Y. Zhang, D.-L. Zhan, Z.-Y. Huang, Z.-N. Lin, Y.-C. Lin, BNIP3L-Dependent Mitophagy Promotes HBx-Induced Cancer Stemness of Hepatocellular Carcinoma Cells via Glycolysis Metabolism Reprogramming, Cancers. 12 (2020) 655. https://doi.org/10.3390/cancers12030655.

[23] M.A. Dragovich, A. Mor, The SLAM family receptors: Potential therapeutic targets for inflammatory and autoimmune diseases, Autoimmun. Rev. 17 (2018) 674–682. https://doi.org/10.1016/j.autrev.2018.01.018.

[24] Z. Feng, H. Qian, K. Li, J. Lou, Y. Wu, C. Peng, Development and Validation of a 7-Gene Prognostic Signature to Improve Survival Prediction in Pancreatic Ductal Adenocarcinoma, Front. Mol. Biosci. 8 (2021) 676291. https://doi.org/10.3389/fmolb.2021.676291.

[25] C. Carbone, G. Piro, M. Fassan, A. Tamburrino, M.M. Mina, M. Zanotto, P.J. Chiao, C. Bassi, A. Scarpa, G. Tortora, D. Melisi, An angiopoietin-like protein 2 autocrine signaling promotes EMT during pancreatic ductal carcinogenesis, Oncotarget. 6 (2015) 13822–13834. https://doi.org/10.18632/oncotarget.2635.

[26] H.-M. Chen, W. van der Touw, Y.S. Wang, K. Kang, S. Mai, J. Zhang, D. Alsina-Beauchamp, J.A. Duty, S.K. Mungamuri, B. Zhang, T. Moran, R. Flavell, S. Aaronson, H.-M. Hu, H. Arase, S. Ramanathan, R. Flores, P.-Y. Pan, S.-H. Chen, Blocking immunoinhibitory receptor LILRB2 reprograms tumor-associated myeloid cells and promotes antitumor immunity, J. Clin. Invest. 128 (2018) 5647–5662. https://doi.org/10.1172/JCI97570.

[27] S. Hashimoto, S. Furukawa, A. Hashimoto, A. Tsutaho, A. Fukao, Y. Sakamura, G. Parajuli, Y. Onodera, Y. Otsuka, H. Handa, T. Oikawa, S. Hata, Y. Nishikawa, Y. Mizukami, Y. Kodama, M. Murakami, T. Fujiwara, S. Hirano, H. Sabe, ARF6 and AMAP1 are major targets of KRAS and TP53 mutations to promote invasion, PD-L1 dynamics, and immune evasion of pancreatic cancer, Proc. Natl. Acad. Sci. U. S. A. 116 (2019) 17450–17459. https://doi.org/10.1073/pnas.1901765116.

[28] L. Sun, X. Zhang, Q. Song, L. Liu, E. Forbes, W. Tian, Z. Zhang, Y. Kang, H. Wang, J.B. Fleming, B.C. Pasche, W. Zhang, IGFBP2 promotes tumor progression by inducing alternative polarization of macrophages in pancreatic ductal adenocarcinoma through the STAT3 pathway, Cancer Lett. 500 (2021) 132–146. https://doi.org/10.1016/j.canlet.2020.12.008.
